# The 25th Anniversary of *Current Issues in Molecular Biology*: Marking This Significant Milestone

**DOI:** 10.3390/cimb46120858

**Published:** 2024-12-19

**Authors:** Madhav Bhatia

**Affiliations:** Department of Pathology and Biomedical Science, University of Otago, Christchurch 8140, New Zealand; madhav.bhatia@otago.ac.nz or madhav1245@gmail.com

*Current Issues in Molecular Biology (CIMB)* (https://www.mdpi.com/journal/cimb) has been in existence since 1999, so this year—2024—represents a major milestone for the journal—its 25th Anniversary. It has been my privilege to serve the journal as its Editor-in-Chief since 2022. Serving as the Editor-in-Chief of *CIMB* is a thoroughly enjoyable experience. My responsibilities in this role include making publication decisions about manuscripts, overseeing the editorial process, managing and upholding peer review guidelines and ethics guidelines, and making scientific decisions regarding the journal’s scope. I am supported by a very capable Editorial Board, Journal Development Editor, the editorial staff, and peer reviewers, as well as the readers of the journal, and I relish every aspect of my role as the Editor-in-Chief.

The journal is broad-based and publishes articles spanning diverse disciplines related to molecular biology. The journal publishes articles on all aspects of molecular biology, from basic mechanisms to practical applications. Leaving the scope of the journal broad is a conscious decision, as we intend to publish articles from different disciplines, as well as articles that are multi-disciplinary in nature. Our goal for the journal is to publish excellent-quality papers in diverse disciplines, under the broad umbrella of molecular biology. With this in mind, the journal continues to evolve and improve, and I am proud of my role, as Editor-in-Chief, in making this possible.

In this regard, I would like to highlight five recent articles that have received attention from the scientific community, either in terms of citations or views.

The first article [1] reviews our current understanding of oxidative stress in type 2 diabetes and its implications, from pathogenesis to lifestyle modifications. Oxidative stress plays a key role in the pathogenesis of diabetes and associated complications. The imbalance between reactive oxygen species production and antioxidant defence mechanisms in the body leads to cellular damage and dysfunction. Chronic hyperglycaemia contributes to increased reactive oxygen species production, further exacerbating oxidative stress. This article [1] summarises, in a concise manner, the wide scope of the genesis of oxidative stress in diabetes, the role of oxidative stress in its complications, and dietary strategies and physical exercise to counter oxidative stress in managing this condition. This article in the field of molecular pathology describes basic research with significant potential in type 2 diabetes in human patients. This excellent article was published as part of the Topic “Oxidative Stress and Mitochondrial Dysfunction in Metabolic and Inflammatory Diseases” and has been cited 107 times.

The second article [2] summarises recent research on ionic homeostasis, osmotic stress regulation, oxidative stress regulation, and plant hormonal responses under salt stress. Soil salinization inhibits plant growth, which can lead to reduced food security and agricultural development. Excessive salt can cause ionic, osmotic, and oxidative stress in plants. Plants maintain ionic homeostasis and stimulate phytohormone signalling pathways by excluding excess salt from their cells, which helps in balancing growth and stress tolerance, and, in turn, enhance their survival. The authors suggest that research achievements in ionic homeostasis, osmotic stress regulation, oxidative stress regulation, and plant hormonal responses under salt stress lay the foundation for a comprehensive understanding of plant salt tolerance mechanisms. This article in the field of molecular plant sciences has significant value in the discipline of agriculture. This is another article that presents a complex subject in an accessible manner, and it has been cited 40 times.

The third article [3] is a research paper that describes synthesis, molecular docking, and dynamic simulation targeting the main protease (Mpro) of new thiazole-clubbed pyridine scaffolds as a potential COVID-19 inhibitor. Many antiviral and chemotherapeutic agents have been found to be ineffective against COVID-19 in the clinic. This article represents a potential novel therapeutic agent against COVID-19. Although much more research is needed before the clinical relevance of this approach could be ascertained, this paper represents a step in the right direction. This paper, combining aspects of molecular biophysics, molecular informatics, and molecular biology, was published as part of the Special Issue “Drug Development and Repositioning Methodology on COVID-19” and has been cited 35 times.

The fourth article [4] highlights the importance of fruit peel extracts and natural products obtained in food industries and their potential biological applications. Fruit by-products are produced in large quantities during industrial processing and may be harmful for the environment. However, there is evidence suggesting that, in some instances, fruit peels may have biological and pharmacological benefits that have not been fully exploited. Fruit peels may, therefore, serve as a useful resource in health-promoting products. This review provides evidence that fruit peels are a rich source of biologically active natural compounds, with potentially significant benefits for human and animal health. This article also suggests investing in the fruit peel processing industry as a pathway in drug discovery. This article, covering the areas of molecular plant sciences, molecular biology, and molecular pharmacology, has attracted significant interest from readers and has been viewed 14,174 times. It has also been cited 25 times.

Finally, I would like to highlight a comprehensive review describing the beneficial effects of red wine polyphenols on human health [5]. Over the years, this subject has generated a great deal of controversy, and there is no clear consensus, which suggests a need to investigate this topic in greater detail. Polyphenols are plant metabolites synthesised during the development of the grape berry in response to stress. They are important constituents of red wines that contribute to their sensory properties and antioxidant activity. In this article, the authors review the relevant literature and describe the impact of red wine polyphenols on human health. In particular, the effects of red wine polyphenols on human health, such as anti-inflammatory, anticarcinogenic, and cardio-protective effects, are discussed. Many studies have been conducted on the health-promoting effects of red wine polyphenols, including cancer chemopreventive activities, neuroprotective effects, and impacts in cardiovascular diseases and in the gut microbiota in humans. In this article, the impact of red wine polyphenols in cardiovascular disease, cancer, the gut microbiota, oral health, and diabetes has been discussed. This article, spanning the areas of molecular pathology, molecular pharmacology, and molecular plant biology, has attracted a high level of interest among readers of *CIMB* and has been viewed 10,235 times and cited 34 times. This article was published as part of the Special Issue “Bioactive Compounds of Natural Products on Metabolic Disorders and Complications”.

These articles represent some of the most viewed or most cited recent articles in *CIMB*. There are several other articles like these, and we are proud of the quality of the work published in the journal. As we celebrate 25 years of *CIMB*, I foresee exciting and intellectually stimulating times ahead for both our journal and the broad discipline of molecular biology. To celebrate the 25th anniversary of *CIMB*, I, along with Prof. Dr. Ru Chih C. Huang and Dr. Hidayat Hussain, am editing a Special Issue entitled, “The 25th Anniversary of *CIMB*: Perspectives in Molecular Biology”. I cordially invite you to contribute to this effort and be part of this journey.

## References

[1] Caturano A., D’Angelo M., Mormone A., Russo V., Mollica M.P., Salvatore T., Galiero R., Rinaldi L., Vetrano E., Marfella R. (2023). Oxidative Stress in Type 2 Diabetes: Impacts from Pathogenesis to Lifestyle Modifications. Curr. Issues Mol. Biol..

[2] Fu H., Yang Y. (2023). How Plants Tolerate Salt Stress. Curr. Issues Mol. Biol..

[3] Alghamdi A., Abouzied A.S., Alamri A., Anwar S., Ansari M., Khadra I., Zaki Y.H., Gomha S.M. (2023). Synthesis, Molecular Docking, and Dynamic Simulation Targeting Main Protease (Mpro) of New, Thiazole Clubbed Pyridine Scaffolds as Potential COVID-19 Inhibitors. Curr. Issues Mol. Biol..

[4] Hussain H., Mamadalieva N.Z., Hussain A., Hassan U., Rabnawaz A., Ahmed I., Green I.R. (2022). Fruit Peels: Food Waste as a Valuable Source of Bioactive Natural Products for Drug Discovery. Curr. Issues Mol. Biol..

[5] Buljeta I., Pichler A., Šimunović J., Kopjar M. (2023). Beneficial Effects of Red Wine Polyphenols on Human Health: Comprehensive Review. Curr. Issues Mol. Biol..

